# Microemulsions as Solubilizers and Penetration Enhancers for Minoxidil Release from Gels

**DOI:** 10.3390/gels7010026

**Published:** 2021-03-03

**Authors:** Miroslava Špaglová, Mária Čuchorová, Martina Čierna, Silvester Poništ, Katarína Bauerová

**Affiliations:** 1 Department of Galenic Pharmacy, Faculty of Pharmacy, Comenius University, Odbojárov 10, SK-832 32 Bratislava, Slovakia; cuchorova@fpharm.uniba.sk (M.Č.); cierna@fpharm.uniba.sk (M.Č.); 2 Centre of Experimental Medicine, Slovak Academy of Sciences, Dúbravská cesta 9, SK-841 04 Bratislava, Slovakia; silvester.ponist@savba.sk (S.P.); katarina.bauerova@savba.sk (K.B.)

**Keywords:** minoxidil, microemulsion, lecithin, gelatin, penetration enhancer, sodium alginate, carrageenan, carbomer, in vitro drug release, ex vivo penetration

## Abstract

Micro- and nanoemulsions are potential drug solubilizers and penetration enhancers through the high surfactant/co-surfactant content. This study aimed to evaluate the influence of minoxidil (MXD) solubilized in the microemulsions (MEs) on drug release by in vitro/ex vivo diffusion through the semi-permeable membrane Spectra/Por^®^ (Spectrum Laboratory, Gardena, CA, USA) and porcine ear skin. Moreover, a residual amount of drug in the skin after ex vivo diffusion was evaluated. The reference ME_R_, lecithin-containing ME_L_, and gelatin-containing ME_G_ were characterized in terms of their size, polydispersity index, density, viscosity, electrical conductivity and surface tension. Based on the in vitro diffusion, it can be argued that ME_L_ slowed down the drug release, while ME_R_ and ME_G_ have no significant effect compared to the sample, in which propylene glycol (PG) was used as a solubilizer. Determination of the residual drug amount in the skin after 6 h of the ex vivo permeation was demonstrated as the most valuable method to evaluate the effectiveness of the ME’s application. The results indicate that the most optimal MXD permeation enhancers in alginate gel were the natural surfactants containing MEs. MXD solubilization in ME_G_ and ME_L_ had caused more than 5% of the drug remaining in the skin, which is almost a 1.5-fold higher amount compared to the reference gel.

## 1. Introduction

Hair has a significant effect on the overall appearance of a person, so disorders manifesting by changes in colour, density, thickness, or structure of the hair are often a reason to seek medical advice or other professional help. Androgenic alopecia (alopecia androgenetica), a determined hair loss, is caused by excessive synthesis of dihydrotestosterone due to pathological conversion from testosterone by 5-alpha reductase [1]. The incidence of androgenic alopecia depends on ethnic and familial factors. Genetic predisposition, age, and androgenic hormones are crucial [2].

U.S. Food and Drug Administration has approved for the treatment of alopecia orally administered finasteride and dermally administered minoxidil (MXD) [3,4]. Previously it was indicated as an oral antihypertensive drug. However, after long-term use (more than one month), patients experienced its main “adverse” effect, namely hypertrichosis. This side effect has been used to benefit people with androgenic alopecia [3,5]. Topically applied MXD stimulates hair growth. It dilates the capillaries in the skin, thus improving the overall blood flow at the site of application [6]. According to other studies, MXD stimulates cell proliferation, inhibits collagen synthesis, and stimulates vascular endothelial growth factor and prostaglandin synthesis [6,7]. Recent research proves that MXD-stimulated secretion of growth factors by adipose-derived stem cells enhances hair growth by promoting dermal papilla proliferation [8]. After topical application, MXD prevents incipient and advanced hormonal hair loss. With long-term application (several months to a year), the production of new terminal hair was observed in 50% to 80% of patients [9]. MXD solutions (2%, *w*/*w*) applied twice a day proved to be therapeutically optimal [10].

More than 40% of newly developed chemical entities (potential drugs) are poorly soluble in water, compromising their bioavailability. There are some traditional approaches to drug solubilization (particle size reduction, pH adjustment, the addition of surfactant/co-surfactant, etc.), while microemulsions (MEs) and self-emulsifying systems present novel approaches [11].

MEs form spontaneously in an adequate ratio of appropriately selected ingredients. They contain an aqueous phase, an oil phase, a surfactant, and when needed a co-surfactant. MEs are thermodynamically stable and optically isotropic [12,13]. Due to a continuously fluctuating interface, they are characterized as perfect drug solubilizers [14]. These colloidal systems are used either as vehicles alone or as drug solubilizers in drug dosage forms, e.g., gels [15]. Many studies confirm their positive effect on drug penetration or transdermal delivery [16,17,18,19,20].

As Maitra et al. [21] have reported, one of the shortages of commercially available MXD formulations is the high content of alcohol used to achieve better drug solubility and skin permeability. If the function of alcohols was substituted by MEs, it could decrease the irritating effect and even enhance the penetration. Some experimental studies investigated MEs or nanoemulsions (NEs) effect on MXD delivery from themselves as the pure vehicles, but they have not yet been studied as solubilizers incorporated in hydrogels. The benefits of MEs/NEs or other penetration enhancer techniques to facilitate MXD permeability are described more in the discussion. Salim et al. [22] have provided a detailed overview of the possibilities to support MXD delivery in topical products, in which they had reported, among others, that many natural ingredients, e.g., phospholipids, polysaccharides, and hyaluronic acid, are useful enhancers in MXD delivery from topical formulations.

In this study, we aimed to evaluate the influence of the solubilization and the enhancer capabilities of the reference microemulsion (ME_R_) and its modifications containing the natural surfactants, lecithin (ME_L_), and gelatin (ME_G_), on the release of sparingly water-soluble MXD. The comparison was performed by in vitro diffusion through a semi-permeable membrane, by ex vivo permeation through porcine ear skin, and finally by determination of the residual drug amount in the skin after previous ex vivo permeation. In conventional MXD solutions, propylene glycol (PG) is used to dissolve the drug [23]; therefore, it was used as the solubilizer in the comparative gel. MXD gels represent a more advantageous dosage form compared to the solutions through higher adhesion to the skin. MXD solutions are perceived as more demanding to apply, and besides, flow quickly from the scalp. Ultimately, the study results on the residual drug amount determination in the skin speak in favour of the MEs.

Minoxidil solubilization in a microemulsion system is a unique tool for incorporation of poorly water-soluble drugs in hydrophilic vehicles, such as hydrogels. As the results of the drug deposition have shown, the microemulsions improve not only the drug solubility but also the drug retention in the skin, mainly caused by the microemulsions with the content of the natural surfactants, lecithin and gelatin. The findings can be applied in the development of topical antialopetic MXD formulation.

## 2. Results and Discussion

### 2.1. Preparation and Physical Characterization of Microemulsions

If a ME is to be used as a carrier or a solubilizer of a poorly soluble drug, the system containing hydrophilic and lipophilic parts in equilibrium is preferred. The hydrophilic continuous phase of the ME (type *o*/*w*) is responsible for its easy transport through the hydrated skin together with the drug loaded in the oil droplets. After a reorganization of the lipid structure in the stratum corneum, the drug is released into the lipophilic environment due to the higher affinity of the ME’s lipophilic regions to the lipid layers of the skin [24].

To reduce the content of polysorbate 80, which may irritate the skin at higher concentration, the natural surfactants (lecithin and gelatin) were added, so that hydrophilic-lipophilic balance (HLB) was maintained (the composition see in Table 1).

The comparison was performed by determination of the basic physical parameters, which are given in Table 2. The droplet size of the internal phase corresponds to the criterion [25,26], i.e., is less than 200 nm. The average droplet size of the inner phase ranged from 127.6 nm to 158.3 nm. Polydispersity index (PDI) was less than 0.283 in all cases, which means that they are almost uniform systems. The viscosity and density increased slightly after the addition of gelatin, while the influence of lecithin is not significant. Although the pH of the skin is below 5.0 [27], topical preparations with a pH in the range from 4 to 7 are physiologically harmless and non-irritating. All prepared MEs had a pH in the range of these values. However, while the MEs were used only as drug solubilizers, not as the topical vehicles, the pH of the final dermal product was decisive. It was from 6.2 to 7.0 in all gels. Based on the results of electrical conductivity, it can be concluded that these MEs are of the *o*/*w* type.

The HLB values may predict the action of surfactant(s), e.g., HLB <10 is suitable for the ME type *w*/*o*, while HLB >10 for the ME type *o*/*w* [28]. The HLB value surfactant/co-surfactant mixture (polysorbate 80/isopropyl alcohol; 1:1) in ME_R_ is 11.24 and only slightly changed with the addition of the natural surfactants. The stable emulsions or MEs contain surfactant(s) mixture with the HLB equal to 10 [29]. There is also other experimental evidence that similar to the emulsions the ME type can be predicted based on the HLB value of surfactant/co-surfactant/oil mixture. If the HLB is from 3 to 6, the most probably it is the ME type *w*/*o*, while the HLB value from 8 to 18 predicts the ME type *o*/*w* [13,30]. Finally, the same is confirmed by the HLB calculation [29] of surfactant(s)/co-surfactant/oil mixture (ME_R_ ~7.79, ME_L_ ~7.76, ME_G_ ~7.85). The calculated HLB values [31] were out of this range but closer to 8.

### 2.2. Solubility of Minoxidil

Drug solubility as one of the most important physical parameters affects considerably the drug bioavailability, the rate of its release, and finally the therapeutic efficacy of the pharmaceutical product. The consideration of drug solubility is one of the primary steps in drug formulation. Also, for the selection of an ME’s components, the investigation of the drug solubility in individual solvents is a crucial point [16,32].

MXD is sparingly soluble in water but freely soluble in methanol and propylene glycol [33]. The drug solubility in a ME is not simple to evaluate visually by kinetic testing. The simple solubility equilibrium test was used to compare the solubilizing capacity of the MEs such as the solvents contained in them. The principle is that an excess of the drug is stirred for 50 to 72 h in a small volume of a solvent/vehicle at room temperature, then after centrifugation and filtration, the drug concentration is determined [34]. The results are recorded in Table 3. They indicate that MXD was dissolved to the highest extent in the MEs, which can be explained by the synergistic effect of the solubilizing abilities of the surfactant, the co-surfactant and the oil phase contained.

### 2.3. Characterization of the Gels

Based on the optimal appearance and relatively close values of the structural viscosity, gels containing the natural gelling agents, sodium alginate (ALG) and carrageenan (CRG), were selected for the in vitro drug release studies. ALG gel had the structural viscosity (η) 4.37 Pa·s and CRG 4.66 Pa·s at the shear rate (D) 6.45 s^−1^. In the pharmaceutical and cosmetic industry, polymers of polyacrylic acid (carbomers, CRB) are widely used as gelling agents. In the study, CRB gel was selected as the comparative synthetic gel base. Its structural viscosity (η) was 15.13 Pa·s at the shear rate (D) 6.45 s^−1^.

### 2.4. Stability

Insignificant changes in transmittance, as well as the viscosity of the MEs after three months, were observed. The ME-based gels showed no phase separation or precipitation after centrifugation that indicate they are stable during their investigation. The 3-month stability test was not performed because fully aqueous gels were prepared without the co-solvents traditionally used for MXD solubilization or any preservatives to avoid other interaction or unwanted influences on the ME. Therefore, it is expected that the hydrogels are stable no longer than one month at 4–8 °C.

### 2.5. The In Vitro Diffusion Study

**Figure 1:**
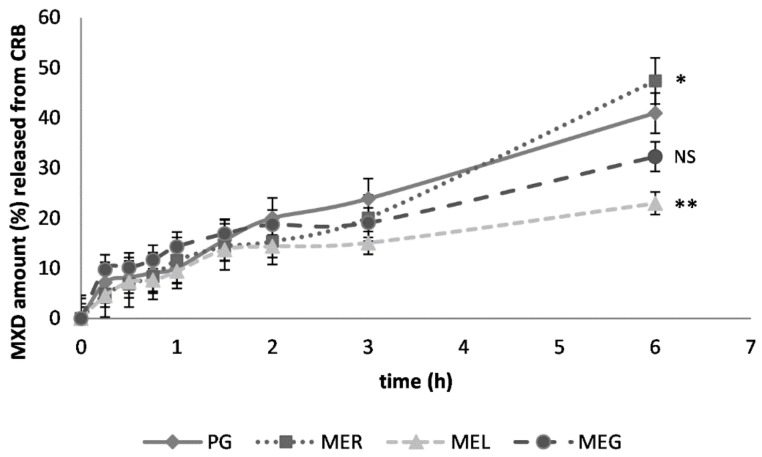
In vitro diffusion of MXD from carbomer gel (CRB) through semi-permeable membrane; previous solubilization in propylene glycol (PG), reference ME_R_, lecithin-containing ME_L_ or gelatin-containing ME_G_. The cumulative MXD amount (%), a mean ± standard error of the mean (SEM), *n* = 4. Significantly different data against reference (PG) are at *p* < 0.05 (*), *p* < 0.01 (**), non-significantly different data (NS) are at *p* > 0.05.

The cumulative amount of MXD released from CRB gel after 6 h during the in vitro diffusion study was the highest after the drug solubilization in ME_R_ (Figure 1). The presence of lecithin or gelatin in the ME slowed down the drug release. The cumulative amounts of MXD were determined to be more than 50% lower after 6 h of the drug diffusion caused by ME_L_ and 32% lower by the effect of ME_G_. The diffusion profiles corresponding to the drug solubilization in PG, ME_R_ and ME_L_ varied slightly during the first one and a half hours.

**Figure 2:**
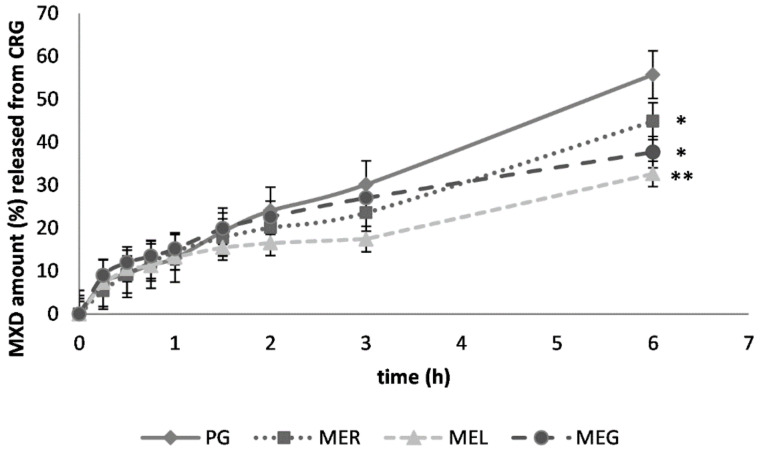
In vitro diffusion of MXD from carrageenan gel (CRG) through semi-permeable membrane; previous solubilization in propylene glycol (PG), reference ME_R_, lecithin-containing ME_L_ or gelatin-containing ME_G_. The cumulative MXD amount (%), a mean ± SEM, *n* = 4. Significantly different data against reference (PG) are at *p* < 0.05 (*), *p* < 0.01 (**), non-significantly different data (NS) are at *p* > 0.05.

In CRG gel, PG acted as the most effective drug solubilizer as the in vitro results have shown. The drug solubilization in the ME_R_ had caused that the cumulative amount of drug released after 6 h was reduced by 20%. The presence of lecithin, as well as gelatin in the ME, caused the slowdown of drug release even more (Figure 2). Statistically significant differences in the drug release after 6 h were confirmed after the drug solubilization in each microemulsion (ME_R_, ME_L_, ME_G_).

**Figure 3:**
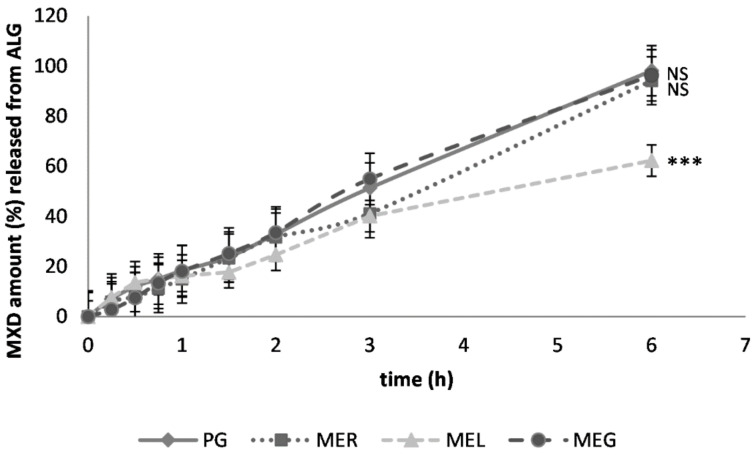
In vitro diffusion of MXD from sodium alginate gel (ALG) through semi-permeable membrane; previous solubilization in propylene glycol (PG), reference ME_R_, lecithin-containing ME_L_ or gelatin-containing ME_G_. The cumulative MXD amount (%), a mean ± SEM, *n* = 4. Significantly different data against reference (PG) are at *p* < 0.005 (***), non-significantly different data (NS) are at *p* > 0.05.

The cumulative MXD amounts released after 6 h were the highest when ALG gel was used as the dermal vehicle. They ranged from 94.09% to 98.09%. The only exception caused the drug solubilization in ME_L_, which resulted in a reduction by 34% of the drug released finally. The diffusion profiles for the ME_R_-consisting gel and ME_G_-consisting gel were almost identical (Figure 3). Based on the Student’s *t*-test results, only the effect of the drug solubilization in ME_L_ caused a significant difference in drug release compared to the reference sample (with PG).

### 2.6. The Ex Vivo Permeation Study

The retardation of the drug release into the dissolution medium does not necessarily mean that the ME is ineffective as the penetration enhancer. It could even be argued that if the drug is not released to a large extent into the dissolution medium, it remains in the skin, and increases the therapeutic potential of topically administered drugs, which target the skin, similarly to the treatment of androgenic alopecia. Therefore, the in vitro diffusion studies were supplemented by the ex vivo permeation studies through the porcine ear skin, which pursued two main objectives. Firstly, we determined the rate of drug diffusion through the skin, respectively, to determine the dermal vehicle with a maximum cumulative amount of drug diffused into the dissolution medium through the skin. Secondly, we determined the drug deposition in the skin after 6 h of the ex vivo permeation study.

**Figure 4:**
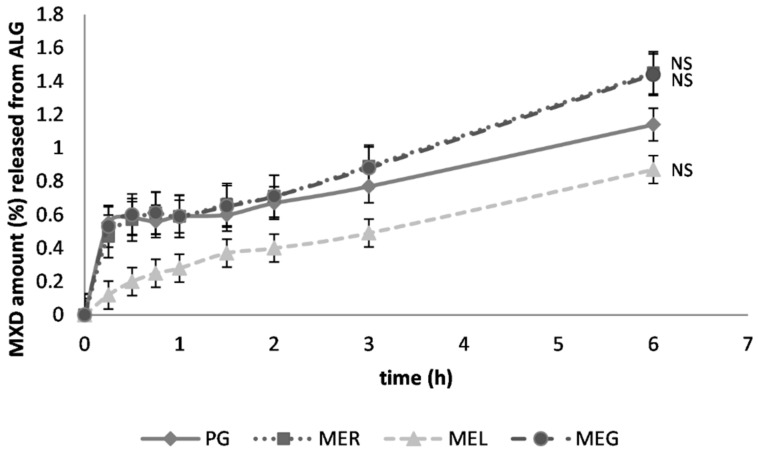
Ex vivo permeation of MXD from sodium alginate gel (ALG) through porcine ear skin; previous solubilization in propylene glycol (PG), reference ME_R_, lecithin-containing ME_L_ or gelatin-containing ME_G_. The cumulative MXD amount (%), a mean ± SEM, *n* = 4. Non-significantly different data (NS) against reference are at *p* > 0.05.

For the ex vivo permeation study the gel was used, from which MXD was released to the largest extent during the in vitro liberation study, i.e., sodium alginate gel. A relatively low percentage of drug permeated to the dissolution medium through the skin (Figure 4). The highest cumulative drug amount was found after the drug solubilization in ME_R_ and ME_G_ (only 1.45%, 1.44%), which could be interpreted as a relatively low risk of the drug absorption into the bloodstream. Due to the drug solubilization in ME_L_, the permeated drug amount was further reduced by approximately 40%. The amounts of MXD permeated through the animal skin were relatively low and the effect of the drug solubilization in the MEs was not evaluated as statistically significant (Figure 4).

**Figure 5:**
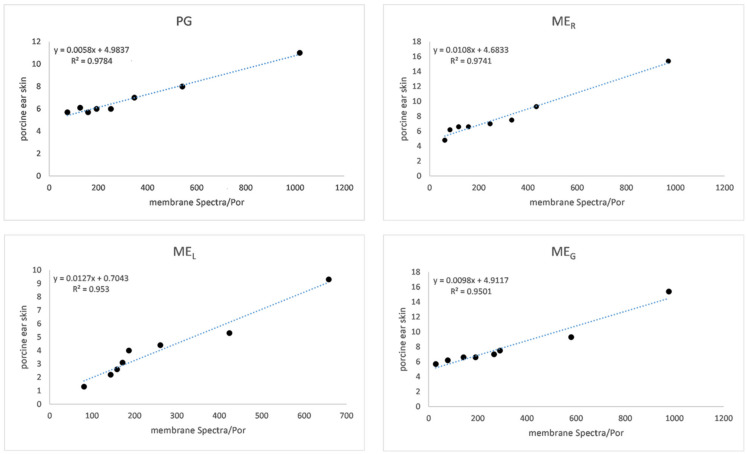
The correlation between in vitro and ex vivo MXD release (10^−2^ μg·cm^−2^) from sodium alginate gel through membrane Spectra/Por^®^ and porcine ear skin. Propylene glycol (PG), reference ME_R_, lecithin-containing ME_L_, gelatin-containing ME_G_.

Data summarized in Table 4 show that the highest flux was achieved using alginate gel under in vitro conditions. Fluxes are approximately the same after solubilization in PG, ME_R_ and ME_L_. The lowest flux during in vitro drug release was recorded from the ME_L_-containing carbomer gel. However, the lowest flux during ex vivo permeation was found from alginate gel containing as the drug solubilizers PG and ME*_G_*. An enhancement ratio, if higher than 1, expresses the positive effect of the ME over the comparative sample (with PG) was recorded only in the ME_R_-based carbomer gel under in vitro conditions and in alginate gel caused by all MEs under ex vivo conditions. Similarly, the highest permeability coefficients (C_P_) were recorded for the same formulations. The ex vivo C_P_ values were 10^−2^ lower, which could be done due to nature and permeation feature of the barrier used (synthetic membrane versus animal skin). As expected, the semi-permeable membrane Spectra/Por^®^ (Spectrum Laboratory, Gardena, CA, USA) is a more permeable barrier to drug diffusion even due to its composition (natural cellulose obtained from short cotton) and thickness (60–65 μm). From animal models, porcine skin is preferred for ex vivo permeation studies because of many similarities to human skin [35]. Although it does not appear, between in vitro and ex vivo drug release data (μg·cm^−2^) is a high correlation (see in Figure 5, expressed as R^2^) [36].

Finally, the results of the in vitro diffusion study could be interpreted also as the passage of the drug through the stratum corneum representing the least permeable layer of the skin to the lower layers of the epidermis and the dermis representing by the dissolution medium. Therefore, in this in vitro model situation depicting drug permeation, it would not be possible to monitor drug absorption into the systemic circulation. The synthetic membrane may simulate the intercellular lipids of the stratum corneum during in vitro diffusion studies, whereas animal skin used during ex vivo permeation studies has imitated transdermal drug release with the subsequent absorption to the bloodstream represented by dissolution media.

### 2.7. Drug Release Kinetics

Drug release kinetics were compared through the coefficient of determination (R^2^) expressed for the regression line of three kinetic models. Table 5 shows the values of the coefficient (R^2^) for the individual kinetic models. The values indicate that the drug release during in vitro diffusion studies follows mostly zero-order kinetic (in rare cases Higuchi model), whereas ex vivo drug release follows either zero- or first-order kinetic. Generally, the drug release rate, which follows zero-order kinetics, is independent of the drug concentration, i.e., it is not influenced by increasing or decreasing drug concentration. Conversely, when the drug is released by first-order kinetics, the drug permeation rate is greatly affected by the drug concentration in the skin. In contrast, the Higuchi model characterizes the kinetics of drug release from sustained release formulations or transdermal systems [37]. Therefore, it may be assumed that the natural surfactants, lecithin and gelatin, in the MEs are responsible for the changes in MXD release kinetics from carbomer, partially also carrageenan gel.

### 2.8. Drug Deposition in the Skin

**Figure 6:**
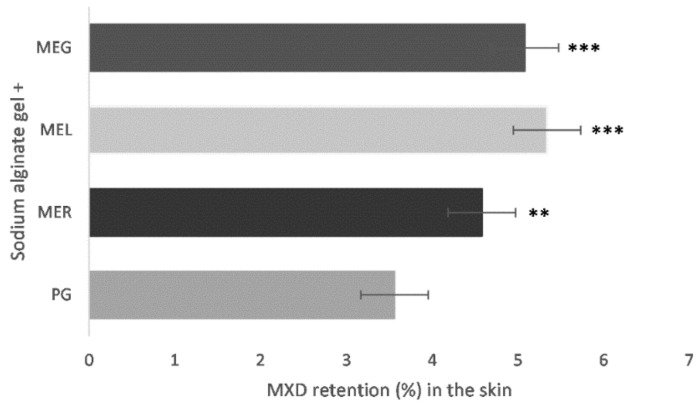
MXD deposition in the skin after ex vivo permeation from sodium alginate gel (ALG); solubilization in propylene glycol (PG), reference ME_R_, lecithin-containing ME_L_ or gelatin-containing ME_G_. Significantly different data against reference (PG) are at *p* < 0.01 (**) and *p* < 0.005 (***).

As Figure 6 shows, the highest drug deposition amounts (5.34% ± 0.71, 5.08% ± 0.54) were determined in the skin pieces, where gels containing MEs with the natural surfactants, lecithin and gelatin, were applied. Based on the statistical analysis of the data obtained, it can be stated that the influence of the drug solubilization in MEs on MXD amount remained in the skin compared to the reference (PG) is in all cases statistically significant (*p* < 0.05).

This method appears to be the optimal for evaluation of penetration enhancer’s effectivity. It clearly depicts the enhancer ability to promote drug permeation into the skin. Based on the results, natural surfactants containing MEs appear to be the most effective permeation enhancer for MXD.

In general, the ME can be considered as a composed enhancer system. The synergistic effect of penetration enhancers, a surfactant, a co-surfactant and an oil phase, is responsible for the support of drug delivery to the skin [38]. The MEs are more often investigated as topical vehicles for example, or as ME-based gels, in which gelling agents are added to increase their viscosity and adhesion to the skin [39]. However, in our study, the MEs were used as solubilizers, mainly due to the high content of polysorbate in the MEs. The in vitro and ex vivo drug release study did not confirm the beneficial effect of lecithin-containing ME_L_ on MXD delivery but rather slowed drug release. Therefore, it was unexpected that the drug deposition study has shown significant (*p* < 0.05) enhancement of MXD amount in the skin after the solubilization in the MEs, mainly with the content of the natural surfactants, ME_L_ and ME_G_. The finding does not correlate with the results of in vitro and ex vivo drug release according to which ME_L_ appears to be an ineffective penetration enhancer. Drug retention in the skin depends on many factors; ME particle size, drug solubility in gel components, gel microstructure, the proportion of gel components, gel viscosity, etc. Finally, the drug deposition study appears to be the most valuable method for determination of the enhancer activity.

In the last decade, many experimental studies have focused on the improvement of topical administration of MXD by different strategies: nanoemulsions [40,41], nanovesicles [42], nanostructured lipid carriers [43], electrodynamic microneedles [44], the synergistic effect of other drugs, e.g., tretinoin [45] and others [46]. The study of Maitra et al. [21] brought positive results in terms of MXD enhancement in permeation through the skin (in vitro, ex vivo, in vivo) from ME-based formulation consisting of oleic acid, PEG 600, Span 20 and water. Visual control via in vivo study suggested that hair loss was slowed down significantly after the application of optimised ME-based formulation compared to the alcoholic formulation. The positive effect was attributed to oleic acid and its good moisturising and wetting property. It supported not only permeation of the drug but also the moistening of the hair follicle and the skin. Alginate gel was also used to incorporate a solid inclusion complex consisting of MXD-hydroxypropyl-β-cyclodextrin. Results concerning the improvement of hair growth were similar to MXD solution, but the application of this system showed a certain superiority in terms of target gene expression profile and hair quality [47]. Interesting are also the results of a randomized study in which 32 men aged 18–30 years were given MXD in a ME, solution or placebo for 32 weeks. The best results in terms of hair numbers, thickness and weight were obtained in a group of men, for whom a multimodal ME containing MXD, diclofenac and tea tree oil was applied [48]. Abd et al. [40], in turn, compared the effect of the oil phase on the ex vivo permeation capacity of MXD-loaded nanoemulsions (NEs), one containing oleic acid, the other eucalyptus oil. The permeation through stratum corneum was significantly improved by NEs compared to the control. Whereas eucalyptus oil-containing NE caused higher retention of MXD in the stratum corneum, the oleic acid-containing NE caused more pronounced hair follicle penetration.

Lecithin is commonly used in topical products at a concentration of 0.1% to 1%, which corresponds to lecithin concentration in the ME_L_, so it could be used as a vehicle alone if it did not contain a high concentration of polysorbate 80, which can be irritating to the skin. Therefore, MEs were only used to solubilize the drug. Lecithin is non-irritating and non-sensitizing to human or animal skin. It has been found that by non-occlusive application in the form of liposomes, 30% of lecithin penetrates the murine skin and up to 99% has accumulated in the piglet skin [49]. Therefore, it can be argued that its penetration into the stratum corneum is high. However, the mechanism of action of topically applied liposomes remains unclear. Some authors define them as drug carriers that penetrate the skin intact, the others claim that they act as penetration enhancers that reversibly alter properties of the stratum corneum so that it temporarily becomes more permeable [50]. Most likely, liposomes will deliver the drug to the stratum corneum by fast partitioning, while the vesicles remain in the SC, the drug continues to the deeper skin layers [50,51]. An ATR-FTIR (attenuated total reflection-Fourier transform infrared-spectroscopy) test has demonstrated the same mechanism of the action of lecithin-containing NE on curcumin delivery. Lecithin and oil components remained in the surface layers of the stratum corneum. Also, an ex vivo tape stripping method revealed that the type of oil phase in NE appears to affect the depth of drug penetration into the stratum corneum [52]. The effect of lecithin-containing ME or NE on drug release was monitored in many works e.g., lecithin-containing ME enhanced topical delivery of terconazole through animal skin [53]. Lecithin-linker ME can provide twice the lidocaine penetration and absorption through the skin as a conventional emulsion. The same study demonstrated sustained lidocaine transdermal delivery [54]. Gelatin-stabilized ME-based gels were useful as a topical vehicle for the administration of cyclosporin A. Transdermal drug delivery via the ME-based gels predicts many advantages and minimal side effects compared to oral administration [55]. Generally, the effect of gelatin content in MEs has been investigated only rarely, concerning more the microstructure changes and physical parameters than penetration enhancer capabilities.

Lecithin is a primary component of colloidal vesicles, such as liposomes or transferosomes, has a wide application in modern drug technology. The presumption that the aforementioned vesicles are a perfect drug carrier lies in one or more principles: (1) changes in the ultrastructure of the stratum corneum, leading to improved drug penetration, (2) drug-loaded vesicles are adsorbed onto the stratum corneum and fused to transport the drug through the lipid layer, (3) vesicles penetrate easily through hair follicles, (4) the interaction of the positively charged components of the liposomes and the negatively charged components of the stratum corneum [56,57]. Lecithin’s influence was not negligible even in the prepared ME_L_. Based on the results, it can be concluded that the lecithin-containing ME_L_ is effective not only as of the drug solubilizer but also as the penetration enhancer for MXD, and hypothetically for all poorly water-soluble drugs. It can be assumed that some of the aforementioned principles of liposomal action contribute to improving the penetration enhancer capability of the lecithin-containing ME_L_.

Comparing the in vitro drug release profiles, it might be concluded that lower viscous alginate gel ensured higher drug release. An inverse relation exists between formulation viscosity and drug release possibility [58]. If the drug is not “imprisoned” strongly in a gel structure, it moves and diffuses through a membrane easily. As expected, the synthetic membrane is not a suitable tool for observation an enhancer activity of MEs. However, it is a way to compare the influence of their solubilizing capabilities on in vitro drug release rate. The penetration enhancers modify the nature of stratum corneum and thereby improve drug partitioning to the tissue [59].

## 3. Conclusions

To achieve a reasonable duration and the most favourable therapeutic effect of a drug after topical administration, a suitable dermal vehicle able to deliver the drug into the target is required. MEs as the solubilizers of poorly soluble drugs may help to improve the drug permeation.

By decreasing the content of polysorbate 80 in the ME of known composition due to the addition of natural surfactants, lecithin and gelatin, it was possible to examine another two MEs as the potential penetration enhancers. Both corresponded to the definition of MEs in terms of their appearance, as well as physical properties. The addition of the natural surfactant caused an increase in the droplet size, while the PDI remained roughly the same. The in vitro drug release through a synthetic membrane is not sufficient to evaluate the efficacy of the penetration enhancers. Animal skin models perform the function of the permeation barrier much more effectively. Although the ex vivo permeation studies did not confirm a significant effect of the MEs on the drug release, the skin samples were subsequently subjected to the drug deposition test, which showed that the addition of lecithin and gelatin to the ME had caused an increase in drug accumulation in the skin. Therefore, it can be concluded that the optimal method to compare penetration enhancer efficacy appears to be the determination of the drug deposition in the skin.

## 4. Materials and Methods

### 4.1. Materials

All materials were of analytical grade and used as received without further purification. Alginic acid sodium salt, carrageenan and isopropyl myristate, minoxidil (MXD) were purchased from Sigma Aldrich (Steinheim, Germany). Polysorbate 80, sodium chloride, ethanol (96%) and isopropyl alcohol were purchased from Centralchem (Bratislava, Slovakia). Propylene glycol (PG) was purchased from Interpharm (Bratislava, Slovakia), Carbopol 71G NF from Lubrizol (Cleveland, USA), soybean lecithin from Dimica (ASP, Divina, Slovakia) and gelatin from Valúch (Banská Bystica, Slovakia). Double-distilled water used for the experiments was prepared at the Department of Galenic Pharmacy (Kavalier, Labo SK, Bratislava, Slovakia). Porcine ear skin was acquired from a local slaughterhouse (Stará Huta, Slovakia).

### 4.2. Preparation of the Microemulsions

The MEs were prepared by the conventional titration method. The surfactant (polysorbate 80) and co-surfactant (isopropyl alcohol) were mixed in the ratio of 1:1 (*w*/*w*). The oil phase (isopropyl myristate) was added during continuous stirring until complete dissolution was achieved. Subsequently, distilled water was added drop by drop avoiding this way “over-titration” manifesting by turbidity. The natural surfactants, lecithin and gelatin, were used to slightly decrease the content of polysorbate 80 so that the HLB would be preserved. The composition of the reference ME_R_, as well as the MEs with the content of the natural surfactants, lecithin (ME_L_) and gelatin (ME_G_), is shown in Table 1.

### 4.3. Physical Characterization of the Microemulsions

The prepared MEs were characterized in terms of the inner droplet size and PDI using Zetasizer Nano NS ZEN 3600 (Malvern Instruments, Ltd., Malvern, UK) and analysed by CONTIN method (Malvern software, Malvern Instruments, Ltd., Malvern, UK). Electrical conductivity was measured by conductometer (Conductivity/TDS -CO 3000H, VWR International GmbH, Vienna, Austria), pH value by pH meter (pH/temperature meter HI 991 001, Hanna Instruments, Woonsocket, RI, USA) and viscosity using Ostwald´s capillary viscometer. For each characteristic, three parallel measurements were performed at 25 ± 1 °C.

### 4.4. Solubility of Minoxidil

To compare MXD solubility in the individual hydrophilic components of the MEs (water, isopropyl alcohol, polysorbate 80) and the MEs themselves, the solubility equilibrium test was performed according to the modified method of drug solubility test [60]. MXD (1%; *w*/*w*) was dissolved in the solvent or the ME for 50 h during continuous stirring on an electromagnetic stirrer at the room temperature. Subsequently, the supernatant was separated from the insoluble sediment by centrifugation at 3000 rpm (CompactStar CS4, VWR International GmbH, Vienna, Austria). The supernatant was filtered through a syringe filter (Q-Max^®^ RR, 0.60 μm, Lambda Life, Bratislava, Slovakia). The samples were diluted with double-distilled water and the drug concentration was determined spectrophotometrically (Genesys 10 UV-VIS (ultraviolet–visible), Cambridge, UK) at 280 nm.

### 4.5. Preparation and Characterization of the Gels

As topical vehicles for the drug solubilized in the MEs three gels were used: sodium alginate (ALG), carrageenan (CRG) and carbomer (CRB). The corresponding amount of the gelling agent (see Table 6) was dispersed in half of the required amount of purified water. It was left to swell for 15 min and then refilled with the remaining water. For neutralization of CRB gel, the aqueous solution of sodium hydroxide (10%, *w*/*w*) was used. The composition of the gels is shown in Table 6. The main rheological characteristics of gels were measured by a rotational viscometer (Anton Paar, Rheolab QC, Graz, Austria), pH values were checked by pH/temperature meter HI 991 001 (Hanna Instruments, RI, USA).

### 4.6. Preparation of the Drug-Loaded Gels

MXD (2 %, *w*/*w*) was solubilized in propylene glycol (PG, the comparative sample) or one of the ME systems (ME_R_, ME_L_, ME_G_). Then the gel (CRB/CRG or ALG) was added so that the final ratio of the ME and the gel was 1:4 (*w*/*w*). This weight ratio was evaluated as the most optimal concerning the appearance, as well as the consistency of the samples in the previous studies [61,62].

### 4.7. Stability

The stability of the MEs was carried out comparing the data (transmittance and viscosity) 24 h after preparation and three months after the storage at 25 °C/4 °C. The stability of ME-based gels was evaluated by observation of the phase separation after 30 min of centrifugation at 3,000 rpm (CompactStar CS4, VWR, Vienna, Austria).

### 4.8. In Vitro Diffusion Study

In vitro diffusion studies were performed by the Franz diffusion cell method. The Franz cell as the acceptor chamber consists of the inner chamber filled with a dissolution medium and the external shell with circulating water heated stable to 32 °C. The semi-permeable membrane Spectra/Por^®^ (Spectrum Laboratory, Gardena, CA, USA) was fastened between the donor chamber and the acceptor chamber filled with the double-distilled water up to the brim. The sample (1.2 g of the gel) was carefully transferred to the upper part of the membrane, while the lower one was in direct contact with the dissolution medium (double-distilled water). The diffusion area was 2.27 ± 0.03 cm^2^. The solution (5 mL) from the acceptor chamber was taken to analyse in 0.25, 0.5, 0.75, 1, 1.5, 2, 3, 6 h and replaced with an equal volume of double-distilled water so that *sink* condition was achieved. The concentration (c, μg·mL^−1^) of MXD in the samples was determined spectrophotometrically at 280 nm against double-distilled water. Equation (1) was obtained from the standard curve:

(1) $$c=\left(A-0.031\right)/1.833$$

### 4.9. Ex Vivo Permeation Study

The procedure was performed similarly to in vitro diffusion except that the animal skin was used instead of the semi-permeable membrane. The ears from six-month-old slaughtered pigs were obtained from a slaughterhouse (Stará Huta, Slovakia) without any prior heat treatment. They were stored at −18 °C until the experiment was performed (max. of 2 weeks). After thawing, the skin was rinsed with normal saline solution, degreased and cut into pieces of sufficient size to allow them to be fixed on the donor chambers. The skin thickness was 2.24 mm ± 0.34. The blind test was taken into account when cumulative amounts were determined.

### 4.10. Drug-Release Kinetics

For determination of drug-release kinetics following plots were made: a cumulative percentage of drug release vs. time (zero-order kinetics); log cumulative percentage of drug remaining vs. time (first-order kinetics); a cumulative percentage of drug release vs. square root of time (Higuchi) [63].

### 4.11. Drug Deposition in the Skin

After the ex vivo permeation studies, the rest of the gel was gently but completely removed from the skin surface. The skin pieces were cut into small circles with the area corresponding to the diffusion area of the Franz cell. The skin cuttings were further macerated in propylene glycol for 24 h. Then the solutions with the skin were centrifuged for 30 min at 3000 rpm (CompactStar CS4, VWR, Vienna, Austria) and filtrated through the syringe filter (Q-Max^®^ RR, 0.60 μm, Lambda Life, Bratislava, Slovakia). Finally, the drug concentration in the water-diluted solutions (50-times) was determined spectrophotometrically.

### 4.12. Data Analysis

The MXD cumulative amount (μg·cm^−2^) diffused through an area of 2.27 cm^2^ was plotted against time. Steady-state flux (μg·cm^−2^ h^−1^) was determined from the slope of the linear portion of the cumulative amount vs. time [40]. The permeability coefficients *C_P_* were calculated from flux (*J*) and initial drug concentration (*C_i_*) according to the Equation (2). Enhancement ratio (*ER*) compares the drug flux from the ME-based gel and the reference gel in Equation (3) [38].

(2) $$C_{p}=J/C_{i}$$

(3) $$ER=J\left(me+gel\right)/J{\left(gel\right)}_{\;}$$

### 4.13. Statistical Analysis

The result of the in vitro and ex vivo diffusion studies were expressed as a mean (± SD) of four parallel measurements. The Student’s *t*-test was used to evaluate a statistical difference between the drug release from the ME-containing gels and the comparative gel containing PG as the drug solubilizer. Data were considered significantly different at *p* < 0.05 (*), *p* < 0.01 (**), *p* < 0.005 (***), or non-significantly different (NS) at *p* > 0.05.

## Figures and Tables

**Table 1:** The Composition of the Microemulsions—Reference Microemulsion (ME_R_), Lecithin-Containing Microemulsion (ME_L_), Gelatin-Containing Microemulsion (ME_G_)_._

	ME_R_ (%, *w*/*w*)	ME_L_ (%, *w*/*w*)	ME_G_ (%, *w*/*w*)
Polysorbate 80	26.50	26.50	26.50
Isopropyl alcohol	26.50	26.50	26.50
Isopropyl myristate	16.00	15.25	16.00
Water	31.00	31.00	30.25
Lecithin	-	0.75	-
Gelatin	-	-	0.75

**Table 2:** The Basic Physical Parameters of the Microemulsions.

	ME_R_	ME_G_	ME_L_
Size (nm)	127.6 ± 2.6	158.3 ± 3.2	148.0 ± 3.0
Polydispersity index	0.279 ± 0.006	0.283 ± 0.006	0.244 ± 0.005
Viscosity (mPa·s)	22.44 ± 0.07	26.69 ± 0.02	22.15 ± 0.05
Density (g·cm^−3^)	0.936 ± 0.001	0.940 ± 0.001	0.939 ± 0.001
pH	6.65 ± 0.01	6.09 ± 0.01	6.21 ± 0.00
Conductivity (μS·cm^−1^)	28.00 ± 0.58	17.93 ± 0.69	16.83 ± 0.71
Surface tension (mN·m^−1^)	28.26 ± 0.02	27.98 ± 0.02	27.79 ± 0.03

Reference ME_R_, gelatin-containing ME_G_ and lecithin-containing ME_L_ (A Mean, ± Standard Deviation (SD), *n* = 3).

**Table 3:** The Comparison of Minoxidil (MXD) Solubility Equilibrium in the Solvents and the Microemulsions.

Solvent	Solubility (mg·mL^−1^)
water	0.247
Isopropyl alcohol	0.367
Polysorbate 80	1.098
ME_R_	1.377
ME_L_	1.364
ME_G_	1.214

**Table 4:** Steady-State Flux (J_SS_), Permeability Coefficients (C_P_) and Enhancement Ratio (ER) for In Vitro and Ex Vivo Drug Release from the Gels.

Gel and Solubilizer	J_SS_ (μg·cm^−2^·h^−1^)	C_P_ (10^−5^ In Vitro; 10^−7^ Ex Vivo)	ER
	**In Vitro**		
CRB + PG	0.619	2.58	-
CRB + ME_R_	0.768	3.20	1.24
CRB + ME_L_	0.252	1.05	0.41
CRB + ME_G_	0.365	1.52	0.59
CRG + PG	0.877	3.66	-
CRG + ME_R_	0.635	2.65	0.96
CRG + ME_L_	0.449	1.87	0.96
CRG + ME_G_	0.403	1.68	0.60
ALG + PG	1.678	6.99	-
ALG + ME_R_	1.606	6.69	0.72
ALG + ME_L_	1.614	6.73	0.51
ALG + ME_G_	1.013	4.22	0.46
	**Ex Vivo**		
ALG + PG	0.012	5.00	-
ALG + ME_R_	0.182	7.58	1.52
ALG + ME_L_	0.182	7.58	1.52
ALG + ME_G_	0.012	5.00	1.00

Carbomer (CRB)/Carrageenan (CRG)/Sodium Alginate (ALG) after drug solubilization in propylene glycol (PG), reference ME_R_, lecithin-containing ME_L_, or gelatin-containing ME_G_.

**Table 5:** The Coefficients of Determination (R^2^) for the Drug Release Kinetics.

Gel & Solubilizer	R^2^ (Zero-Order)	R^2^ (First-Order)	R^2^ (Higuchi)
**In Vitro**			
CRB + PG	0.9879	0.8932	0.9829
CRB + ME_R_	0.9790	0.9076	0.9527
CRB + ME_L_	0.9083	0.7447	0.9841
CRB + ME_G_	0.9675	0.8965	0.9775
**In Vitro**			
CRG + PG	0.9968	0.8930	0.9662
CRG + ME_R_	0.9819	0.8152	0.9076
CRG + ME_L_	* 0.9655	0.8813	0.9685
CRG + ME_G_	0.9569	0.8355	* 0.9554
**In Vitro**			
ALG + PG	0.9980	0.8786	0.9471
ALG + ME_R_	0.9943	0.8721	0.9342
ALG + ME_L_	0.9800	0.8733	0.9494
ALG + ME_G_	0.9940	0.7281	0.9749
**Ex Vivo**			
ALG + PG	0.9738	0.9862	0.8744
ALG + ME_R_	0.9835	0.9773	0.9108
ALG + ME_L_	0.9846	0.8277	0.9754
ALG + ME_G_	0.9733	0.9877	0.8768

* close to the highest value.

**Table 6:** The Composition of the Gels; Carbomer (CRB), Sodium Alginate (ALG), and Carrageenan (CRG).

	CRB (%, *w*/*w*)	ALG (%, *w*/*w*)	CRG (%, *w*/*w*)
Carbomer	1.0	-	-
Sodium alginate	-	4.0	-
Carrageenan	-	-	4.0
NaOH (10% sol., *w*/*w*)	4.0	-	-
water	97.0	96.0	96.0

## Data Availability

The data presented in this study are available on request from the corresponding author. The data are not publicly available due to restriction.

## References

[1] Suchonwanit P., Thammarucha S., Leerunyakul K. (2019). Minoxidil and Its Use in Hair Disorders: A Review. Drug Des. Dev. Ther..

[2] Braun-Falco O., Plewig G., Wolff H.H. (2001). Ochorenia vlasov. Dermatológia a Venerológia.

[3] Yum S., Jeong S., Kistm D., Lee S., Kim W., Yoo J.-W., Kim J.-A., Kwon O.S., Kim D.-D., Min D.S. (2017). Minoxidil Induction of VEGF Is Mediated by Inhibition of HIF-Prolyl Hydroxylase. Int. J. Mol. Sci..

[4] Price V.H. (1999). Treatment of Hair Loss. N. Engl. J. Med..

[5] Randolph M., Tosti A. (2020). Oral Minoxidil Treatment for Hair Loss: A Review of Efficacy and Safety. J. Am. Acad. Dermatol..

[6] Messenger A.G., Rundegren J. (2004). Minoxidil: Mechanisms of Action on Hair Growth. Br. J. Dermatol..

[7] Goren A., Naccarato T. (2018). Minoxidil in the Treatment of Androgenetic Alopecia. Dermatol. Ther..

[8] Choi N., Shin S., Song S., Sung J.-H. (2018). Minoxidil Promotes Hair Growth through Stimulation of Growth Factor Release from Adipose-Derived Stem Cells. IJMS.

[9] Ohyama M. (2010). Management of Hair Loss Diseases. Dermatol. Sin..

[10] Tully A.S., Schwartzenberger J., Studdiford J. (2010). Androgenic Alopecia. J. Men’s Health.

[11] Gowardhane A.P., Kadam N.V., Dutta S. (2014). Review on Enhancement of Solubilization Process. Am. J. Drug Discov. Dev..

[12] McClements D.J. (2012). Nanoemulsions versus Microemulsions: Terminology, Differences, and Similarities. Soft Matter.

[13] Tartaro G., Mateos H., Schirone D., Angelico R., Palazzo G. (2020). Microemulsion Microstructure(s): A Tutorial Review. Nanomaterials.

[14] Mathur V., Satrawala Y., Rajput M.S. (2014). Physical and Chemical Penetration Enhancers in Transdermal Drug Delivery System. Asian J. Pharm. (AJP) Free Full Text Artic. Asian J. Pharm..

[15] Mehta D.P., Rathod H.J., Shah D.P., Shah C.N. (2015). A Review on Microemulsion Based Gel: A Recent Approach for Topical Drug Delivery System. Res. J. Pharm. Technol..

[16] Sahle F.F., Metz H., Wohlrab J., Neubert R.H.H. (2013). Lecithin-Based Microemulsions for Targeted Delivery of Ceramide AP into the Stratum Corneum: Formulation, Characterizations, and in Vitro Release and Penetration Studies. Pharm. Res..

[17] Santos P., Watkinson A.C., Hadgraft J., Lane M.E. (2008). Application of Microemulsions in Dermal and Transdermal Drug Delivery. Skin Pharmacol. Physiol..

[18] Singh V., Veerma R., Singh M., Javed A., Sharma H. (2013). Topical Non Steroidal Anti *Inflammatory* Drug (NSAIDs Microemulsions: Rationale, Review and Future Prospective. Asian J. Pharm..

[19] Souto E.B., Doktorovova S., Boonme P. (2011). Lipid-Based Colloidal Systems (Nanoparticles, Microemulsions) for Drug Delivery to the Skin: Materials and End-Product Formulations. J. Drug Deliv. Sci. Technol..

[20] Kumar K.S., Dhachinamoorth D., Saravanan R., Gopal U.K. (2011). Microemulsions as carrier for novel drug delivery: A review. Int. J. Pharm. Sci. Rev. Res..

[21] Maitra M., Goyal A.K., Rath G. (2017). A Novel Approach for Follicular Delivery of Minoxidil for Treatment of Alopecia. J. Drug Deliv. Sci. Technol..

[22] Salim S., Kamalasanan K. (2020). Controlled drug delivery for alopecia: A review. J. Control. Release.

[23] Barbareschi M., Vescovi V., Starace M., Piraccini B.M., Milani M. (2020). Propylene Glycol Free 5% Minoxidil Lotion Formulation: Cosmetic Acceptability, Local Tolerability, Clinical Efficacy and in-Vitro Skin Absorption Evaluations. G. Ital. Dermatol. Venereol..

[24] Bouwstra J.A., Honeywell-Nguyen P.L., Gooris G.S., Ponec M. (2003). Structure of the Skin Barrier and Its Modulation by Vesicular Formulations. Prog. Lipid Res..

[25] Azeem A., Khan Z.I., Aqil M., Ahmad F.J., Khar R.K., Talegaonkar S. (2009). Microemulsions as a Surrogate Carrier for Dermal Drug Delivery. Drug Dev. Ind. Pharm..

[26] Lawrence M.J., Rees G.D. (2000). Microemulsion-Based Media as Novel Drug Delivery Systems. Adv. Drug Deliv. Rev..

[27] Lambers H., Piessens S., Bloem A., Pronk H., Finkel P. (2006). Natural Skin Surface PH Is on Average below 5, Which Is Beneficial for Its Resident Flora. Int. J. Cosmet. Sci..

[28] Ramli S., Chyi K.T., Zainuddin N., Mokhtar W.N.A.W., Abdul Rahman I. (2019). The Influence of Surfactant/Co-Surfactant Hydrophilic-Lipophilic Balance on the Formation of Limonene-Based Microemulsion as Vitamin C Carrier. JSM.

[29] Nollet M., Boulghobra H., Calligaro E., Rodier J.-D. (2019). An Efficient Method to Determine the Hydrophile-Lipophile Balance of Surfactants Using the Phase Inversion Temperature Deviation of CiEj/n-Octane/Water Emulsions. Int. J. Cosmet. Sci..

[30] Alam S., Algahtani M.S., Ahmad M.Z., Ahmad J. (2020). Investigation Utilizing the HLB Concept for the Development of Moisturizing Cream and Lotion: In-Vitro Characterization and Stability Evaluation. Cosmetics.

[31] Guo X., Rong Z., Ying X. (2006). Calculation of Hydrophile–Lipophile Balance for Polyethoxylated Surfactants by Group Contribution Method. J. Colloid Interface Sci..

[32] Niazi S.K., Niazi S.K. (2007). The Scope of Preformulation Studies. Handbook of Preformulation.

[33] Council of Europe (2016). Minoxidil. European Pharmacopoeia, Suppl. 8.6..

[34] Gad S.C. (2008). Pharmaceutical Manufacturing Handbook: Production and Processes.

[35] Jacobi U., Kaiser M., Toll R., Mangelsdorf S., Audring H., Otberg N., Sterry W., Lademann J. (2007). Porcine Ear Skin: An in Vitro Model for Human Skin. Skin Res. Technol..

[36] Nada A. (2018). Comparative Ex Vivo and in Vitro Permeation Kinetics of Tocopherol in Liquid Formulations. Asian J. Pharm. (AJP) Free Full Text Artic. Asian J. Pharm..

[37] Dash S., Murthy P.N., Nath L., Chowdhury P. (2010). Kinetic Modeling on Drug Release from Controlled Drug Delivery Systems. Acta Pol. Pharm..

[38] Špaglová M., Čuchorová M., Bartoníková K., Šimunková V. (2020). Chemical penetration enhancers in topical application and their synergistic combination. Chem. Listy.

[39] Boonme P., Kaewbanjong J., Amnuaikit T., Andreani T., M Silva A., B Souto E. (2016). Microemulsion and Microemulsion-Based Gels for Topical Antifungal Therapy with Phytochemicals. CPD.

[40] Abd E., Benson H., Roberts M., Grice J. (2018). Minoxidil Skin Delivery from Nanoemulsion Formulations Containing Eucalyptol or Oleic Acid: Enhanced Diffusivity and Follicular Targeting. Pharmaceutics.

[41] Cardoso S.A., Barradas T.N. (2020). Developing Formulations for Drug Follicular Targeting: Nanoemulsions Loaded with Minoxidil and Clove Oil. J. Drug Deliv. Sci. Technol..

[42] Kumar P., Singh S., Handa V., Kathuria H. (2018). Oleic Acid Nanovesicles of Minoxidil for Enhanced Follicular Delivery. Medicines.

[43] Wang W., Chen L., Huang X., Shao A. (2017). Preparation and Characterization of Minoxidil Loaded Nanostructured Lipid Carriers. AAPS Pharm. Sci. Tech..

[44] Bao L., Gong L., Guo M., Liu T., Shi A., Zong H., Xu X., Chen H., Gao X., Li Y. (2020). Randomized Trial of Electrodynamic Microneedle Combined with 5% Minoxidil Topical Solution for the Treatment of Chinese Male Androgenetic Alopecia. J. Cosmet. Laser Ther..

[45] Sharma A., Goren A., Dhurat R., Agrawal S., Sinclair R., Trüeb R.M., Vañó-Galván S., Chen G., Tan Y., Kovacevic M. (2019). Tretinoin Enhances Minoxidil Response in Androgenetic Alopecia Patients by Upregulating Follicular Sulfotransferase Enzymes. Dermatol. Ther..

[46] Santos A.C., Pereira-Silva M., Guerra C., Costa D., Peixoto D., Pereira I., Pita I., Ribeiro A.J., Veiga F. (2020). Topical Minoxidil-Loaded Nanotechnology Strategies for Alopecia. Cosmetics.

[47] Tricarico D., Maqoud F., Curci A., Camerino G., Zizzo N., Denora N., Cutrignelli A., Laquintana V., Lopalco A., la Forgia F. (2018). Characterization of Minoxidil/Hydroxypropyl-β-Cyclodextrin Inclusion Complex in Aqueous Alginate Gel Useful for Alopecia Management: Efficacy Evaluation in Male Rat. Eur. J. Pharm. Biopharm..

[48] Sakr F.M., Gado A., Mohammed H., Ismail A.A.N. (2013). Preparation and Evaluation of a Multimodal Minoxidil Microemulsion versus Minoxidil Alone in the Treatment of Androgenic Alopecia of Mixed Etiology: A Pilot Study. DDDT.

[49] Fiume Z. (2001). Final Report on the Safety Assessment of Lecithin and Hydrogenated Lecithin. Int. J. Toxicol..

[50] Peralta M.F., Guzmán M.L., Pérez A.P., Apezteguia G.A., Fórmica M.L., Romero E.L., Olivera M.E., Carrer D.C. (2018). Liposomes Can Both Enhance or Reduce Drugs Penetration through the Skin. Sci. Rep..

[51] Maghraby G.M.M.E., Williams A.C., Barry B.W. (2006). Can Drug-Bearing Liposomes Penetrate Intact Skin?. J. Pharm. Pharmacol..

[52] Vater C., Hlawaty V., Werdenits P., Cichoń M.A., Klang V., Elbe-Bürger A., Wirth M., Valenta C. (2020). Effects of Lecithin-Based Nanoemulsions on Skin: Short-Time Cytotoxicity MTT and BrdU Studies, Skin Penetration of Surfactants and Additives and the Delivery of Curcumin. Int. J. Pharm..

[53] Talaat S.M., Elnaggar Y.S.R., Abdalla O.Y. (2019). Lecithin Microemulsion Lipogels Versus Conventional Gels for Skin Targeting of Terconazole: In Vitro, Ex Vivo, and In Vivo Investigation. AAPS Pharm. Sci. Tech..

[54] Xuan X.-Y., Cheng Y.-L., Acosta E. (2012). Lecithin-Linker Microemulsion Gelatin Gels for Extended Drug Delivery. Pharmaceutics.

[55] Liu H., Wang Y., Han F., Yao H., Li S. (2007). Gelatin-stabilised Microemulsion-based Organogels Facilitates Percutaneous Penetration of Cyclosporin a In Vitro and Dermal Pharmacokinetics In Vivo. J. Pharm. Sci..

[56] Shah S.M., Ashtikar M., Jain A.S., Makhija D.T., Nikam Y., Gude R.P., Steiniger F., Jagtap A.A., Nagarsenker M.S., Fahr A. (2015). LeciPlex, Invasomes, and Liposomes: A Skin Penetration Study. Int. J. Pharm..

[57] Schubert R., Žabka M. (1999). Lipozómy v liekoch. Moderné Lieky vo Farmaceutickej Technológii.

[58] Tas C., Ozkan Y., Okyar A., Savaser A. (2007). In Vitro and Ex Vivo Permeation Studies of Etodolac from Hydrophilic Gels and Effect of Terpenes as Enhancers. Drug Deliv..

[59] Das S., Lee S.H., Chow P.S., Macbeath C. (2020). Microemulsion Composed of Combination of Skin Beneficial Oils as Vehicle: Development of Resveratrol-Loaded Microemulsion Based Formulations for Skin Care Applications. Colloids Surf. B Biointerfaces.

[60] Arora R., Aggarwal G., Harikumar S.L., Kaur K. (2014). Nanoemulsion Based Hydrogel for Enhanced Transdermal Delivery of Ketoprofen. Adv. Pharm..

[61] Špaglová M., Žabka M., Čuchorová M., Starýchová L., Vitková M. (2014). Vplyv kosolventov na liberáciu liečiva indometacínu z karbopolových gélov s obsahom mikroemulzie. Chem. Listy.

[62] Špaglová M., Čuchorová M., Šimunková V., Matúšová D., Čierna M., Starýchová L., Bauerová K. (2020). Possibilities of the Microemulsion Use as Indomethacin Solubilizer and Its Effect on in Vitro and Ex Vivo Drug Permeation from Dermal Gels in Comparison with Transcutol^®^. Drug Dev. Ind. Pharm..

[63] Bohrey S., Chourasiya V., Pandey A. (2016). Polymeric Nanoparticles Containing Diazepam: Preparation, Optimization, Characterization, in-Vitro Drug Release and Release Kinetic Study. Nano Converg..

